# Anaesthetic considerations in metabolic syndrome X

**DOI:** 10.4103/0019-5049.60512

**Published:** 2010

**Authors:** Nitesh Goel, Kapil Gupta, Poonam Bhadoria, Raktima Anand

**Affiliations:** Department of Anesthesia and Intensive Care, Maulana Azad Medical College and Lok Nayak Hospital, New Delhi, India

Sir,

Metabolic syndrome X (insulin resistance syndrome, Reaven's syndrome), according to the new International Diabetes Federation (IDF) 2006[1] definition, requires presence of central obesity (defined as waist circumference with ethnicity-specific values≥90 cm in Indian males or ≥80 cm in females or body mass index >30 kg/m^2^) plus any two of the following four factors:

- Raised triglycerides, ≥150 mg/dL (1.695 mmol/L), or specific treatment for this lipid abnormality
- Reduced high-density lipoprotein cholesterol, ≤40 mg/dL (1.03 mmol/L) in males or ≤50 mg/dL (1.29 mmol/L) in females, or specific treatment for this lipid abnormality
- Raised blood pressure, systolic BP≥130 mm Hg or diastolic BP≥85 mm Hg, or treatment of previously diagnosed hypertension
- Raised fasting plasma glucose, ≥100 mg/dL (5.6 mmol/L), or previously diagnosed type 2 diabetes mellitus

We report a case of a 78-year-old man, weighing 90 kg with a height of 1.7 m, who was scheduled for amputation of middle three digits of the left foot (gangrene). He had diabetes for the last 8 years, hypertension for the last 6 months and productive cough since 4 days. He had an anginal attack 6 months back. He was on tab. amlodipine, monotrate, aspirin and inj. insulin for the last 6 months.

He was conscious, oriented and his vitals were PR: 84/min; RR: 14/min; BP: 160/92 mm Hg; and he was afebrile. There were coarse crepitations bilaterally in the chest. His investigations were, haemoglobin: 7 g/dL; blood sugar (fasting): 144 mg/dL; serum sodium: 128 mmol/L; serum potassium: 4 mmol/L; serum triglycerides: 3 mmol/L; urine albumin: positive; chest radiograph: bilateral opacities and ECG: T-wave inversion in leads V2 and V5.

The patient had taken his morning dose of antihypertensive medications. In the operation theatre, monitoring of ECG, SpO_2_ and (Non-invasive blood pressure) NIBP was started, and an 18-G I.V. cannula was secured. His preoperative blood sugar was 140 mg/dL. Insulin infusion and 5% dextrose were started to maintain blood sugar at 120-180 mg/dL.

Under all aseptic precautions, using a peripheral nerve stimulator (Organon, Switzerland), left-sided ankle block was given with 7-mL 1% Lignocaine and 17-mL 0.25% Bupivacaine. Adequate sensory block was achieved. The patient was given oxygen by ventimask (FiO_2_ -0.4). He remained haemodynamically stable intraoperatively and postoperatively.

The patient was diagnosed with metabolic syndrome X due to presence of body mass index 31 kg/m^2^, diabetes, hypertension and hypertriglyceridaemia. Anaesthetic implications of all these co-morbidities were considered.

In view of diabetes mellitus, the patient was evaluated for cervical spine involvement, peripheral neuropathy and autonomic neuropathy.[2] Prophylaxis against aspiration (due to gastroparesis) was used. Obese patients can have an obstructed airway, and there is difficulty in giving regional blocks and securing intravenous access. Hyponatraemia emia leads to poor myocardial contractility, severe hypotension and increased sensitivity to nondepolarizing muscle relaxants.

General anaesthesia was not administered in this patient because of poor chest condition and presence of hyponatraemia. Diabetic peripheral neuropathy presenting after an epidural block may mimic an anaesthetic complication of epidural block.[3] Spinal block in an uncontrolled hypertensive may cause a detrimental fall in blood pressure. Hence peripheral nerve block was considered as the safest option.

Although ultrasound-guided nerve block would have been a better choice,[4] yet its unavailability in most institutes and requirement of special expertise are the limitations. In these situations, peripheral nerve stimulator still plays an ideal role.
